# Allelic Imbalance in TOR1A mRNA Expression in Manifesting and Non-Manifesting Carriers of the GAG-Deletion

**DOI:** 10.1155/2012/985260

**Published:** 2012-09-03

**Authors:** Ioanna A. Armata, Andreas I. Diplas, Laurie J. Ozelius, Pullanipally Shashidharan

**Affiliations:** ^1^Department of Neurology and Radiology Massachusetts General Hospital and Program in Neuroscience, Harvard Medical School, Boston, MA 02114, USA; ^2^Department of Preventive Medicine, Mount Sinai School of Medicine, New York, NY 10029, USA; ^3^Department of Genetics and Genomic Sciences, Mount Sinai School of Medicine, New York, NY 10029, USA; ^4^Department of Neurology, Mount Sinai School of Medicine, New York, NY 10029, USA

## Abstract

Early onset dystonia (EOD) is associated with a 3bp-(ΔGAG) in-frame deletion in the TOR1A gene, which encodes for torsinA. Carriers of the mutant (ΔGAG) allele can either develop or escape a dystonic phenotype (*~*30% penetrance). The expression ratio of the two alleles could be important for the manifestation or prevention of the disease since wild-type (WT) torsinA is thought to have protective function. Absence of an antibody discriminating WT from ΔE torsinA has precluded the determination ΔE and WT torsinA levels in manifesting and nonmanifesting carriers. We performed quantitative analysis of TOR1A allele expression in manifesting (MC) and nonmanifesting (NMC) carriers using quantitative allele-specific PCR (qASPCR) to determine the levels of mutant versus WT torsinA mRNA. The technique described showed high degree of specificity in detecting the two alleles. The present study represents the first comprehensive analysis of biallelic expression of the TOR1A gene in lymphoblast and brain samples from patients and NMC relatives. We demonstrate that mRNA is transcribed from both the WT and ΔGAG allele in peripheral and neural tissues with a trend for increased expression of the ΔGAG allele compared to the WT in carriers regardless of their phenotype and thus cannot account for the reduced penetrance.

## 1. Introduction

Most autosomal genes are transcribed from both alleles except those regulated by genomic imprinting [1]. However, nonimprinted autosomal genes can also demonstrate unequal expression of their two alleles due to *cis* acting polymorphisms in their promoter, enhancer, or other regulatory regions linked to one of the two alleles, selectively affecting its transcription and/or mRNA stability/processing, including splicing and turnover [2, 3]. In fact, it has been estimated that among human brain expressed genes, approximately 20% exhibit unequal expression of the two alleles [2–4]. This phenomenon is termed “allelic imbalance,” and examples of genes showing allelic imbalance include the serotonin transporter (SLC6A4) [5], the mu opioid receptor (*OPRM1*) [6], and the multidrug resistance polypeptide 1 (MDR1, *ABCB1*) [7]. 

Allelic imbalance in gene expression is of particular importance in carriers of a mutant allele associated with a genetic disease. Early onset dystonia (EOD) is associated with a 3 bp-(ΔGAG) in-frame deletion in the *TOR1A* gene [8], which encodes for torsinA [9]. Individuals heterozygous for the mutant (ΔGAG) allele can either develop or escape a dystonic phenotype (~30% penetrance) [10]. The expression ratio of the two alleles could be important for the manifestation or prevention of the disease since wild-type (WT) torsinA is thought to be a protective chaperon, forming homo-hexamers, which are interrupted by the presence of mutant (ΔE) protein [11, 12]. Absence of an antibody discriminating WT from ΔE torsinA has so far only allowed determination of total torsinA levels in manifesting and nonmanifesting carriers.

In the present study, we performed quantitative analysis of *TOR1A* allele expression in manifesting (MC) and nonmanifesting (NMC) carriers using quantitative allele-specific PCR (qASPCR), a method previously published by Chen et al. [13]. The method relies on a frequent single nucleotide polymorphism (SNP) residing within the transcribed region of a gene and the use of a set of two allele-specific primers, which differ only in their end nucleotide corresponding to that SNP (e.g., abcdeC and abcdeT, for a coding SNP being C/T). The primer completely matching one allele is more efficient than the mismatched primer, giving rise to discrete PCR growth curves. Thus, qASPCR allows quantitative analysis of allelic mRNA abundance in samples heterozygous for that particular SNP, which serves as a “readout polymorphism.” In the present study, we used the ΔGAG mutation as a “readout polymorphism” in order to discriminate and quantify separately the expression of each allele. Such analysis allowed us to examine whether NMCs express more of the WT message as compared to the mutant at a ratio rescuing the dystonic phenotype or conversely MCs expressing increased ratio of the mutant compared to the wild-type torsinA mRNA. 

## 2. Experimental Methods

**Figure 1:**
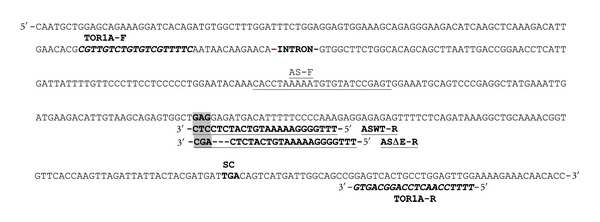
Primers used in allele-specific PCR assays: partial sequence of the 4th and 5th exons of human torsinA is shown and their intronic sequence is indicated by “-INTRON-.” The primer pair (TOR1A-F and TOR1A-R) used to generate a 327 bp human TOR1A fragment from RNA is shown in bold italics. The allele-specific pairs of primers used to discriminate between the WT and ΔE *TOR1A* alleles are underlined. The same allele-specific forward primer is used (AS-F) with two different reverse primers, one matching the WT allele with 3′ ending at CTCCTC (ASWT-R) and the other matching the mutant allele, missing a 303 bp-GAG and thus with a 3′ ending at CGACTC (ASΔE-R). The last three nucleotides, which are different between the two reverse primers, are highlighted in grey; the dashes represent the 3 nucleotides deleted in the ASΔE-R primer. The stop codon (SC) TGA is also indicated in bold. All reverse primers are shown under their corresponding forward sequence in reverse complement orientation.

We designed two allele-specific reverse primers, which differ in their three 3′ end nucleotides, corresponding to the site of deletion, termed ASWT-R and ASΔE-R, and a common forward primer AS-F (Figure 1). The specificity and affinity of the primers was first established by performing ASPCRs on DNA samples isolated from: (1) a control individual (homozygous for the WT allele-WT/WT) expecting the ASWT-R primer to be more efficient, (2) from a *TOR1A *carrier (heterozygous, WT/ΔGAG-TA) expecting the ASWT-R and ASΔE-R primers to be equally efficient, and (3) from a bacterial plasmid expressing human ΔGAG-TA, since there are no individuals homozygous for the ΔGAG allele, expecting the ASΔE-R primer to be more efficient. ASPCRs were performed using a hot-start thermostable Taq DNA polymerase (AmpliTaq Gold) in 1x buffer (50 mM Tris pH 7.5, 50 mM KAc, 2% glycerol, 1× BSA), 0.2 mM dNTPs (A, U, G, C), 4 mM MgAc_2_, 0.2 *μ*M ASWT-R or ASΔE-R primer, 0.2 *μ*M AS-F primer, 1.25 Units of AmpliTaq Gold, 1× SYBR Green, 20 ng/*μ*L DNA template, up to 20 *μ*L with H_2_O, at 95°C for 10 min, 40 cycles 95°C for 30 sec, 65°C for 30 sec, 72°C for 30 sec. All allelic imbalance assays were performed in triplicates in 384 well plates, in the Roche Lightcycler.

The Cp values arising from the PCR growth curves from amplification of DNA from individuals that are either homozygous WT/WT or heterozygous WT/ΔGAG as well as ΔGAG bacterial DNA, using the allele specific set of primers AS-F and ASWT-R or AS-F and ASΔE-R, are shown in Table 1. ΔCp is the difference between the Cp values for the same template run with the two allele-specific primer sets. The results indicate that both ASWT-R and ASΔE-R primers amplify the heterozygous WT/ΔGAG DNA with almost the same efficiency (Ct = 23.33 and 23.13, resp.). However, when using DNA from a noncarrier (WT/WT), the ASWT-R primer is more efficient (Cp = 22.34 cycles) than the ASΔE-R (Cp = 29.55), giving a negative cycle difference (ΔCp) of −7.21 (fold increase 2^−7.21^) indicating increased expression of the wildtype over the mutant allele. On the contrary, the ASΔE-R primer is more efficient (Cp = 7.60) than the ASWT-R (Cp = 19.47) when ΔGAG-TA-plasmid DNA is used, giving a positive ΔCp = 11.87 (fold increase 2^11.87^) indicating increased expression of the mutant over the wild-type allele. Based on that, we concluded that our AS-PCR conditions and the set of primers used could efficiently discriminate between the two alleles of a *TOR1A* carrier.

To determine the levels of expression of the ΔGAG and WT allele in peripheral tissues, we performed qASPCRs on RNA samples isolated from lymphoblasts of five manifesting (MC, 1 to 5) and five nonmanifesting (NMC, 6 to 10) *TOR1A* carriers. RNA was isolated from the samples using the Trizol reagent (Sigma-Aldrich) and treated with Turbo DNAse-free (Ambion) according to manufacture's instructions. 400 ng/*μ*L of DNAse treated RNA was reverse transcribed using a two-step RT-PCR with oligo-dT primers and SuperScript II (Stratagene) in a 20 *μ*L reaction. To generate cDNA the pair of exon-specific primers TOR1A-F and TOR1A-R and Platinum Taq DNA polymerase (Invitrogen) were used to amplify a 327 bp human *TOR1A* fragment flanking the GAG deletion (Figure 1). The cDNA products were diluted to 10^8^, and 2 *μ*L were used for qASPCRs; for each cDNA product two separate reactions were set up: one using the AS-F/ASWT-R set and another using the AS-F/ASΔE-R set of allele-specific primers (Figure 1). Each reaction was repeated in triplicate, with the mean value calculated (MCp) for each pair of primers and then subtracted to yield the difference, ΔCp, between the Cp values of each primer (Cp value for AS-F/ASWT-R minus Cp value for the AS-F/ASΔE-R set of primers) for a particular cDNA sample. As an internal control, to correct for differences due to primer efficiency within each experiment [14], we included a genomic DNA sample from a carrier (WT/ΔGAG) and the ΔCp for the genomic DNA sample was used to normalize the ΔCp value of each cDNA sample. Finally, since a cycle difference reflects double product, the fold increase was expressed as power of 2. QASPCRs were repeated three times and Table 2 shows one representative experiment. As discussed above, the set of allele-specific primers for DNA template from a carrier (WT/ΔGAG) gave very similar Cp values in qASPCRs (Table 1). Likewise, the Cp values obtained using RNA samples from a carrier should be similar unless there is differential expression of one over the other allele. In all samples examined, we obtained positive ΔCps due to slightly lower Cp values for the ΔGAG relative to the WT allele indicating increased mutant allele expression; as shown in Table 2, the ΔCp values varied from 0.70 to 0.21 in MCs and 0.58 to 0.28 in NMCs. 

## 3. Results

Allelic imbalance can be a general phenomenon whereby a particular gene exhibits unequal expression of its two alleles in all tissues. Alternatively, it can be a tissue-specific phenomenon due to transcriptional regulation mechanisms directing unequal expression of the two alleles in a particular tissue. Thus, we expanded our analysis to postmortem brain tissue from one *TOR1A* manifesting and one nonmanifesting carrier (obtained from University of Maryland, Baltimore through its NICHD Brain and Tissue Bank for Developmental Disorders). Total RNA from two different brains regions, globus pallidum (GP) and substantia nigra (SN) (Table 3), was isolated from each brain, the RNA was reverse transcribed, the cDNA generated, and the qASPCRs were performed as described above for the lymphoblast samples. Consistent with what we observed in lymphoblasts, for each brain region, we detected a lower Cp value for the ΔGAG allele indicating increased expression of the ΔGAG over the WT allele (Table 3). Moreover, this increase was detected in both the MC and NMC brains and in both brain areas examined varying from 0.82 to 1.02 (Table 3), levels that are higher than even the greatest increase in expression seen in the lymphoblasts (Table 2).

Of note, the integrity of the RNA used to perform qASPCRs was analyzed by agarose gel to detect the presence of the two ribosomal RNA subunits and assess the amount of degradation (data not shown). Since mRNA is rather labile in autopsy materials, there was a considerable degree of degradation, which varied among the two different brains and two different areas. This could limit comparison between different brain samples, but the ratio of allelic mRNA abundance within each brain sample is expected to be less strongly affected if not at all. Thus, the different degree of degradation between samples should have little or no impact on our analysis since the comparison is between the two alleles within the same sample.

## 4. Discussion 

The present study represents the first comprehensive analysis of biallelic expression of the *TOR1A* gene in lymphoblast and brain samples from patients and NMC relatives. We have shown that mRNA is transcribed from both the WT and ΔGAG allele in peripheral and neuronal tissues with a trend for increased expression of the ΔGAG allele compared to the WT in carriers regardless of their phenotype and thus cannot account for the reduced penetrance. An SNP within the TOR1A gene, D216H, has been associated with penetrance in several studies [15, 16]. This SNP is within a large linkage disequilibrium block encompassing both the TOR1A and TOR1B genes. Combining our finding of no difference in allele expression between MC and NMCs with the overrepresentation of the 216H allele in NMCs suggests that this SNP must also be affecting penetrance through a structural rather than expression mechanism, and by extrapolation, genetic factors located elsewhere in the genome and/or unidentified environmental factors are responsible for the reduced penetrance of EOD dystonia.

Our analysis was limited to one MC and one NMC brain sample because there are very few brains available from patients with a mutation in the *TOR1A *gene. Furthermore, the clinical data associated with the brains in the brain bank is limited, and it is well documented that unless relatives of a proband are examined by a movement disorder specialist, many can go undiagnosed; therefore, we cannot be 100% sure that the NMC really did not manifest dystonia [15].

 Regardless, the data from the brain samples were consistent with what we found in lymphoblast that is, in all samples, the ΔGAG allele was expressed higher level than the WT allele. However, we did note that the trend for increased ΔGAG mRNA levels is higher in brain compared to lymphoblast samples that could partially explain why the GAG deletion selectively affects the CNS. In fact, the two brain areas that were available for examination are thought to play an important role in the pathophysiology of EOD; GPi is the target region for deep brain stimulation in EOD patients [16], while torsinA is highly enriched in the dopaminergic neurons of the SN pars compacta [17], where torsinA promotes DA release while torsinA-ΔE inhibits it [18]. Increased levels of mutant torsinA in those critical brain areas can have serious implications in dopaminergic signaling. However, as other brain regions were not available, further study is required to determine if the increased expression of the mutant allele is a regional or general occurrence. Although the studies reported here are not completely conclusive, the technique and the data provided can be used for further exploration when additional tissues and cell culture samples become available from EOD patients.

## Figures and Tables

**Table 1:** Affinity of allele-specific primers for the wildtype (WT) and the ΔGAG allele of TOR1A.

DNA template	Primers	Cp	ΔCp
1(a) WT/WT	AS-F	22.34	−7.21
	ASWT-R		
1(b) WT/WT	AS-F	29.55	
	ASΔE-R		
2(a) WT/ΔGAG	AS-F	23.33	0.20
	ASWT-R		
2(b) WT/ΔGAG	AS-F	23.13	
	ASΔE-R		
3(a) ΔGAG	AS-F	19.47	11.87
	ASWT-R		
3(b) ΔGAG	AS-F	7.60	
	ASΔE-R		

WT/WT-homozygous for the normal allele; WT/ΔGAG-TOR1A carrier heterozygous for the mutant allele; ΔGAG-plasmid construct expressing only the mutant allele; AS-F, ASWT-R, ASΔE-R: allele-specific set of primers (described in Figure 1); Cp: values from PCR growth curves; ΔCp: Cp difference between the two allele-specific primers (Cp for wildtype minus Cp for mutant) for the same DNA template.

**Table 2:** Expression ratio of WT to ΔGAG TOR1A in lymphoblasts from MC and NMC.

			Lymphoblasts			
			RNA → cDNA samples			
Samples WT/ΔGAG	Phenotype	Detected allele	MCp ± S.D	ΔCp	Corrected ΔCp	Fold expression
1	MC	WT	27.64 ± 0.22	0.56	0.70	2^−0.70^
		ΔGAG	27.18 ± 0.13			
2	MC	WT	24.71 ± 0.08	0.38	0.52	2^−0.52^
		ΔGAG	24.33 ± 0.16			
3	MC	WT	24.87 ± 0.08	0.21	0.35	2^−0.35^
		ΔGAG	24.66 ± 0.06			
4	MC	WT	25.87 ± 0.15	0.36	0.50	2^−0.50^
		ΔGAG	25.51 ± 0.25			
5	MC	WT	27.93 ± 0.13	0.07	0.21	2^−0.21^
		ΔGAG	27.87 ± 0.29			
6	NMC	WT	26.58 ± 0.16	0.39	0.53	2^−0.53^
		ΔGAG	26.19 ± 0.04			
7	NMC	WT	24.8 ± 0.08	0.14	0.28	2^−0.28^
		ΔGAG	24.67 ± 0.41			
8	NMC	WT	29.79 ± 0.17	0.31	0.45	2^−0.45^
		ΔGAG	29.49 ± 0.33			
9	NMC	WT	28.53 ± 0.21	0.44	0.58	2^−0.58^
		ΔGAG	28.09 ± 0.35			
10	NMC	WT	26.93 ± 0.10	0.29	0.43	2^−0.43^
		ΔGAG	26.63 ± 0.19			
Genomic DNA		WT	26.06 ± 0.14	−0.14	0	2^0^
WT/ΔGAG		ΔGAG	26.2 ± 0.48			

WT/WT: homozygous for the normal allele; WT/ΔGAG-TOR1A: carrier heterozygous for the mutant allele; AS-F, ASWT-R, ASΔE-R: allelic-specific set of primers (Figure 1); MC: manifesting carrier; NMC: non-manifesting carrier; MCp ± S.D: mean Cp values of triplicates for each reaction; ΔCp: MCp for WT minus MCp for mutant allele primer for each sample; corrected ΔCp: mean ΔCp of each RNA sample minus ΔCp of heterozygous genomic DNA sample.

**Table 3:** Expression ratio of WT to ΔGAG TOR1A in brains from MC and NMC.

				Brain samples			
				RNA → cDNA samples			
Brain	Area	Phenotype	Detected allele	MCp ± S.D.	ΔCp	Corrected ΔCp	Fold expression
1	GP	M	WT	24.28 ± 0.20	0.83	1.02	2^−1.02^
			ΔGAG	23.45 ± 0.19			
	SN		WT	22.72 ± 0.22	0.63	0.82	2^−0.82^
			ΔGAG	22.72 ± 0.32			

2	GP	NM	WT	20.3 ± 0.28	0.64	0.83	2^−0.83^
			ΔGAG	19.65 ± 0.20			
	SN		WT	19.29 ± 0.17	0.72	0.91	2^−0.91^
			ΔGAG	18.56 ± 0.13			

	Genomic DNA		WT	25.36 ± 0.18	−0.19	0	2^0^
	WT/ΔGAG		ΔGAG	25.55 ± 0.07			

WT/WT: homozygous for the normal allele; WT/ΔGAG-TOR1A: carrier heterozygous for the mutant allele; AS-F, ASWT-R, ASΔE-R: allelic specific set of primers (Figure 1); MC: manifesting carriers; NMC: non-manifesting carrier; GP: globus pallidus; SN: substantia nigra; MCp ± S.D.: mean Cp values of triplicates for each reaction; ΔCp: MCp for WT minus MCp for mutant allele primer for each sample; corrected ΔCp: Mean ΔCp of each RNA sample minus ΔCp of heterozygous genomic DNA sample.
